# Subjektives Gefühl digitaler Ausgegrenztheit

**DOI:** 10.1007/s00391-023-02170-7

**Published:** 2023-03-13

**Authors:** Alexander Seifert

**Affiliations:** Hochschule für Soziale Arbeit, Fachhochschule Nordwestschweiz, Riggenbachstrasse 16, 4600 Olten, Schweiz

**Keywords:** Sozialer Druck, Internet, Techniknutzung, Soziale Teilhabe, Schweiz, Social pressure, Internet, Technology use, Social participation, Switzerland

## Abstract

**Hintergrund:**

Nicht nur jüngere, sondern zunehmend auch ältere Menschen leben heute in einer Welt, in der digitale Technologien ihren Alltag maßgeblich begleiten. Dennoch nutzen gerade ältere Personen seltener die neuesten Technologien. Fühlen sich ältere Menschen dadurch im Vergleich zu jüngeren Personen besonders ausgeschlossen? Um diese Fragen zu beantworten, wurde anhand einer Bevölkerungsbefragung bei Personen ab 18 Jahren das Empfinden digitaler Exklusion gemessen.

**Material und Methoden:**

Das Datenmaterial stammt aus einer Schweizer Befragung (*n* = 1604) von Personen im Alter von 18 bis 98 Jahren. Die Datenerhebung erfolgte als standardisierte Onlinebefragung und wurde mit einer optionalen telefonischen Befragung kombiniert.

**Ergebnisse:**

Ein Teil der Personen unter und über 65 Jahren fühlt sich jeweils sozial ausgrenzt, da er nicht immer die aktuellen Alltagstechnologien beherrscht. Innerhalb der Gruppe der 18- bis 64-Jährigen haben 3,6 % ein sehr starkes Exklusionsempfinden, und bei der älteren Gruppe (65 bis 98 Jahre) beträgt dieser Wert 5,5 %, womit ältere Personen eher zur Gruppe der Personen mit sehr starken Gefühlen digitaler Exklusion gehören. Gleichzeitig zeigt die multivariate Zusammenhangsanalyse, dass der Einfluss des Alters durch andere Variablen (Einkommen, Technikeinstellung) nivelliert wird.

**Schlussfolgerung:**

Auch wenn die digitale Transformation voranschreitet, gibt es bei der Techniknutzung weiterhin Ungleichheiten, die sich in Exklusionsgefühlen äußern können. Neben der Frage, welche älteren Personen Technik nutzen oder nicht, sollte in Zukunft die Frage des subjektiven Empfindens der Ausgrenzung stärker berücksichtigt werden.

## Abstract (en)

**Background:**

Digital technologies play an important role in people’s everyday lives. This applies not only to younger people but also increasingly more to older people. Nevertheless, older persons in particular use the latest technologies less frequently. For this reason, do older people feel particularly excluded compared to younger people? To answer this question, the perception of digital exclusion was measured based on a population survey of people aged 18 years and older.

**Material and methods:**

The data were collected with a survey (*n* = 1604) of Swiss individuals aged 18–98 years. The survey was conducted as a standardized online survey and was combined with an optional telephone survey.

**Results:**

According to the survey results, some people under and over 65 years of age currently feel socially excluded because they cannot always master current everyday technologies. Among the group aged 18–64 years old 3.6% feel a very strong sense of exclusion, and among the older group (65–98 years) 5.5%, which means that older persons tend to belong to the group of persons with very strong feelings of digital exclusion; however, the multivariate correlation analysis showed that the influence of age was levelled out by other variables (income and attitude toward technology).

**Conclusion:**

Although digital transformation is progressing there are still inequalities in technology use, which can manifest in feelings of exclusion. In addition to the question of which older individuals use or do not use technology, the issue of subjective feelings of exclusion should be given greater consideration in the future.

Im Alltag werden Informationen, Dienstleistungen und Services zunehmend nur noch digital angeboten – die aktuelle Coronapandemie hat diese Entwicklung noch einmal verstärkt. Doch fühlen sich gerade ältere Personen digital abgehängt? Dies war die Leitfrage für näher vorzustellende Analyse auf Grundlage einer Befragung der Schweizer Gesamtbevölkerung ab 18 Jahren.

## Hintergrund und Fragestellung

Nicht erst durch die vermehrte Nutzung von Onlineangeboten zur Kontaktaufrechterhaltung während der Kontaktbeschränkungen der COVID-19-Pandemie ist der Zugang zur digitalen Welt und zu ihren Angeboten eine wichtige Prämisse für die soziale Teilhabe in der heutigen Gesellschaft [1]. Diese zunehmende digitale Transformation zeigt sich u. a. im Nutzungsanstieg moderner Informations- und Kommunikationstechnologien (IKT) wie dem Internet oder sprachgesteuerten Assistenzen. Studien aus der Schweiz zeigen z. B. einen Anstieg der Nutzung des Internets oder des Smartphones bei Personen ab 65 Jahren von 2010 bis 2020 [13]. Dennoch ist aus der gerontologischen Forschung bekannt, dass auch heute noch Formen einer digitalen Spaltung zwischen jüngeren (unter 65 Jahre) und älteren Altersgruppen (über 65 Jahre) bestehen [3, 8, 13]. Darüber hinaus hat die COVID-19-Pandemie noch einmal deutlich gemacht, dass gerade jene älteren Menschen, die wenig Erfahrungen mit modernen IKT haben, bei den zunehmenden Beschränkungen des physischen Kontakts nicht selbstverständlich bzw. automatisch auf eine Kontaktaufrechterhaltung über das Internet umstellen konnten. Sie waren der Gefahr einer doppelten Ausgrenzung ausgesetzt: einerseits durch Beschränkung des physischen Kontakts und anderseits durch fehlende Kompensation mithilfe digitaler Lösungen zur Kontaktaufrechterhaltung [14]. Erste Autoren [5] berichten bereits von einem „digitalen Push“ durch die COVID-19-Pandemie, jedoch sind die Ergebnisse bisher gemischt [5], und es bedarf weiterer Vergleichsstudien. Aber auch andere Veränderungen im Schweizer Alltag machen die digitale Transformation deutlich. Hierzu zählt beispielsweise die Mitte 2022 vollzogene Umstellung auf die Begleichung von öffentlichen Rechnungen mithilfe des QR-Codes und nicht mehr eines Einzahlungsscheins, die Verwendung von Online-Banking-Lösungen oder das Verschwinden von lokalen Post- und Bankstellen sowie Ticketautomaten für den öffentlichen Verkehr im ländlichen Raum. Diese Veränderungen bringen die Notwendigkeit mit sich, über „digitale Kompetenzen“ [17] zu verfügen, um digital geprägte Dienstleistungen und Services im Alltag nutzen zu können und nicht von diesen Informationen und Angeboten ausgeschlossen zu sein [6]. Hill et al. [7] haben bereits 2015 eine kumulative, sich selbst verstärkende Spirale, in der die digital Versierten immer mehr integriert und die digital Nichtversierten immer mehr isoliert werden, festgestellt.

Unklar bleibt jedoch, wie digital ausgeschlossen (subjektiv exkludiert) sich ältere Personen (ab 65 Jahren) insgesamt vor der COVID-19-Pandemie fühlten und inwieweit sich dieses Exklusionsgefühl zu dem von jüngeren Personen (18 bis 64 Jahre) unterscheidet. Bisherige Studien (z. B. [8]) haben vorwiegend die Nutzungsunterschiede bezüglich diverser Technologien dieser Altersgruppen untersucht, jedoch nicht das subjektive Gefühl der digitalen Exklusion (SDE). Vor diesem Hintergrund interessierten in der vorliegenden Studie die Intensität und die Prädiktoren des SDE in der Allgemeinbevölkerung. Mit SDE wird ein subjektives Gefühl der Ausgrenzung aus der technologisch-geprägten Gesellschaft verstanden; also die Wahrnehmung, dass man selbst nicht mehr mit den technischen Dingen (Voraussetzungen) des Alltags Schritt halten kann [15]. In einer Schweizer Studie des Jahres 2019 [15] gaben 14,1 % der befragten Personen ab 65 Jahren, die das Internet nicht nutzten, an, dass sie sich manchmal aufgrund dieser digitalen Abstinenz von der Gesellschaft ausgeschlossen fühlen. Diese Ergebnisse konzentrieren sich jedoch nur auf ältere Personen (65 Jahre und älter). Somit ist kein Altersgruppenvergleich möglich. In der vorliegenden Studie wird – angelehnt an dieser Schweizer Studie – davon ausgegangen, dass das Alter einer Person neben anderen soziodemografischen Variablen und Einstellungen zu Technologien dazu beiträgt, SDE vorherzusagen.

## Studiendesign und Untersuchungsmethoden

### Datenerhebung

Vom 29.10.2019 bis zum 17.12.2019 wurden 1604 Personen ab 18 Jahren in allen Sprachregionen der Schweiz befragt. Zum Einsatz kam eine Onlinebefragung, ergänzt durch Telefoninterviews mit Personen, die keinen Internetanschluss haben oder das Internet nicht selbst nutzen. Es wurde ein standardisierter Fragebogen mit geschlossenen Fragen verwendet, der ins Deutsche, Französische und Italienische, die 3 Landessprachen der Schweiz, übersetzt wurde. Eine einfache Zufallsstichprobe der ständigen Wohnbevölkerung der Schweiz im Alter von 18 Jahren und älter wurde aus dem Stichprobenrahmen (Registerdatensatz) des Bundesamts für Statistik gezogen. Es wurden postalische und personalisierte Einladungen sowie 2 schriftliche Erinnerungsschreiben verschickt. Es gab keine Einschränkungen bezüglich des Höchstalters, der aktuellen Internetnutzung, der Nationalität oder der Wohnform der befragten Personen. Die durchschnittliche Interviewdauer für die beiden Befragungsmethoden betrug 41,8 min. Es wurde eine Nettoausschöpfungsrate von 30,5 % erreicht. In Tab. 1 sind die Merkmale der Stichprobe aufgeführt. Das Alter der Befragten reichte von 18 bis 98 Jahren, mit einem Durchschnitt von 51,5 Jahren (SD ± 17,86 Jahre).

**Table 1:** 

Merkmal	Kategorien	Anzahl (*n*)	Anteil (%)
Geschlecht	Frauen	834	52,0
	Männer	770	48,0
Altersgruppen (Jahre)	18–34	328	20,4
	35–54	568	35,4
	55–64	266	16,6
	65–74	260	16,2
	75–98	182	11,3
Bildungsstand	Bis Sekundarstufe I	163	10,5
	Sekundarstufe II	963	61,8
	Tertiärstufe	433	27,8
	Keine Angabe	45	–
Haushaltseinkommen	Bis CHF 4000	218	16,0
	CHF 4001 bis 8000	563	41,2
	Über CHF 8000	584	42,8
	Keine Angabe	239	–
Wohnregion	Ländlich	413	25,7
	Nichtländlich	1191	74,3
Internetnutzung	Online	1517	94,7
	Offline	85	5,3

### Messinstrumente

Das SDE war die abhängige Variable in den Analysen und wurde anhand einer Adaptation der Skala zum Gefühl der Ausgrenzung von Bude und Lantermann [2] gemessen. Diese Skala besteht aus 5 Aussagen: (1) Ich habe Angst, den Anschluss zu verpassen. (2) Ich habe das Gefühl, andere Menschen haben mich abgeschrieben. (3) Ich werde ausgegrenzt. (4) Ich habe das Gefühl, gar nicht richtig zur Gesellschaft zu gehören. (5) Ich habe das Gefühl, im Grunde gesellschaftlich überflüssig zu sein. Die Befragten wurden gebeten, auf einer 5‑stufigen Likert-Skala (1: „trifft überhaupt nicht zu“ bis 5: „trifft voll und ganz zu“) anzugeben, inwieweit jede Aussage auf sie zutrifft. Eingeleitet wurden diese Aussagen mit folgendem Vorwort:

„Denken Sie nun einmal an die heutigen technischen/digitalen Möglichkeiten wie das Internet oder die Selbstbedienungsautomaten, die heute den Menschen zur Verfügung stehen. Wenn Sie nun an diese zunehmende Digitalisierung des Alltags denken, inwieweit würden die nachfolgenden Aussagen auf Sie zutreffen?“

Eine Faktorenanalyse der Antworten auf die oben genannten 5 Aussagen ergab eine einfaktorielle Lösung (Tab. 2). Auf diese Weise wurde der SDE-Wert berechnet (M = 1,76, SD ± 0,817). Höhere Werte spiegeln ein stärkeres SDE wider. Cronbachs α für die Skala beträgt 0,884.

**Table 2:** 

Aussage	Skala	Einfaktorlösung	Gruppenunterschiede					
	M (± SD)	Faktorladungen	Frau	Mann	18 bis 64 Jahre	65 bis 98 Jahre	Onliner	Offliner
Ich habe Angst, den Anschluss zu verpassen	2,05 (± 1,102)	0,726	2,07	2,02	1,99**	2,19**	2,05*	2,25*
Ich habe das Gefühl, gar nicht richtig zur Gesellschaft zu gehören	1,72 (± 0,975)	0,872	1,72	1,72	1,70	1,79	1,70***	2,16***
Ich werde ausgegrenzt	1,69 (± 0,952)	0,854	1,68	1,70	1,62***	1,86***	1,65***	2,32***
Ich habe das Gefühl, im Grunde gesellschaftlich überflüssig zu sein	1,68 (± 0,958)	0,852	1,63*	1,74*	1,65*	1,77*	1,65***	2,15***
Ich habe das Gefühl, andere Menschen haben mich abgeschrieben	1,61 (± 0,886)	0,851	1,59	1,63	1,60	1,63	1,59**	1,88**
*Gesamtskala „SDE“*	*1,76 (±* *0,817)*	*–*	*1,75*	*1,76*	1,72**	1,85**	1,73***	2,15***

Aussagen sortiert nach Mittelwert. Skala (1: „trifft überhaupt nicht zu“ bis 5: „trifft voll und ganz zu“)

Signifikanztest (t-Test): *p < 0,05, **p < 0,01, ***p < 0,001

Es wurde eine Reihe von Prädiktorvariablen berücksichtigt, die in früheren Untersuchungen [15, 16] ermittelt wurden. Dazu gehören die folgenden soziodemografischen Variablen: Geschlecht (1: weiblich und 0: männlich); Alter (Jahre); Bildung (1: Primar-, 2: Sekundar- und 3: Tertiärstufe); monatliches Haushaltseinkommen (1: weniger als 4000 Schweizer Franken [CHF], 2: 4001–8000 CHF und 3: mehr als 8000 CHF); Wohnsituation (1: lebt allein und 0: lebt nicht allein) und Wohngegend (1: ländliche Region und 0: nichtländliche Region). Daneben wurde die Internetnutzung berücksichtigt (1: On-, 0: Offliner). Die folgenden Aspekte der subjektiven Technologieadaptivität – (a) wahrgenommener adaptiver Nutzen, (b) technologiebezogenes Zielengagement und (c) wahrgenommene Sicherheit der Technologie – wurden mit einer kurzen 9‑Item-Version [16] des „Subjective Technology Adaptivity Inventory“ [9] bewertet, bei der die befragten Personen die Items auf einer 5‑Punkte-Skala beantwortet haben (1: „stimme nicht zu“ bis 5: „stimme absolut zu“).

### Statistische Analysen

Neben deskriptiven Darstellungen der Verteilungen wurden Vergleiche zwischen den Altersgruppen berücksichtigt. Zusätzlich wurde eine lineare Regressionsanalyse durchgeführt. Fehlende Werte wurden listenweise eingeschlossen.

## Ergebnisse

### Ermittelte Skalenwerte

Wie anhand von Tab. 2 zu sehen ist, ergibt sich über alle befragten Personen ab 18 Jahren ein Mittelwert von 1,76 für die SDE-Skala, wobei der Mittelwert für 18- bis 64-Jährige 1,72 und für Personen ab 65 Jahren 1,85 beträgt.

Betrachtet man die Personen, die auf der SDE-Skala sehr hohe Werte von 3,5–5 aufweisen, als Gruppe, befinden sich 4,1 % Personen mit einem sehr hohen SDE im Datensatz. In der Gruppe der 18- bis 64-Jährigen beträgt dieser Prozentsatz 3,6 % und in der älteren Gruppe (65 bis 98 Jahre) 5,5 %, womit ältere Personen eher zur Gruppe derer mit sehr hohen Exklusionsgefühlen gehören. Eine bivariate Korrelation zwischen Alter und SDE ergibt einen signifikanten Zusammenhang (Pearsons r 0,068, *p* 0,007).

Im Vergleich zwischen Frauen und Männern ergeben sich keine statistisch signifikanten Unterschiede für die Gesamtskala SDE.

**Figure 1:**
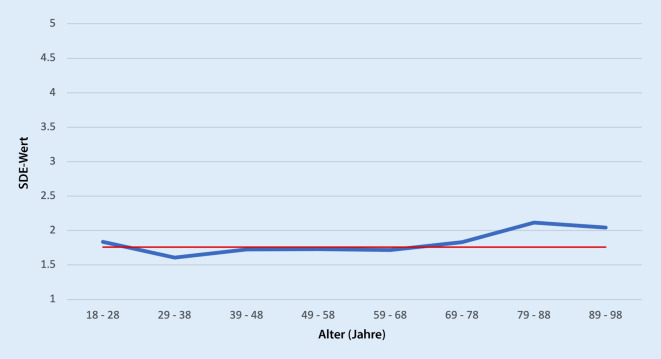

Werden nun die Altersgruppen noch stärker ausdifferenziert, bestätigt sich (Abb. 1) der Unterschied zwischen den jüngeren und älteren Altersgruppen, wobei die Altersgruppe der 29- bis 38-Jährigen die niedrigsten und die älteste Gruppe (75+) die höchsten Werte aufweisen. Jedoch wird bereits visuell deutlich, dass diese Unterschiede auf der SDE-Skala nicht sehr markant ausfallen. Nur das Exklusionsempfinden bei den über 70-Jährigen sticht heraus.

### Einflussfaktoren

Um herauszuarbeiten, inwieweit soziodemografische Merkmale und Technikeinstellungen die SDE-Gesamtskala beeinflussen, wurde eine lineare Regression berechnet (Tab. 3). Werden sowohl standarddemografische Variablen als auch die Techniknutzung (On‑/Offliner) und die Technikeinstellungen berücksichtigt, lassen sich als statistisch signifikante Prädiktoren nur das Einkommen und die wahrgenommene adaptive Nutzenabwägung benennen. Personen mit höherem Einkommen und Personen, die mehr Nutzen in neuen Technologien sehen, fühlen sich weniger digital ausgegrenzt aus der Gesellschaft. Im Gesamtmodell trägt das Alter nicht mehr zur Erklärung bei.

**Table 3:** 

Prädiktoren	Bivariate Einzelregressionen b	Gesamtmodell b	95 %-Konfidenzintervall	β
Alter	0,003**	0,001	−0,002–0,003	0,016
Frau (Ref. Mann)	−0,017	−0,078	−0,160–0,003	−0,050
Bildung	−0,165***	−0,071	−0,151–0,008	−0,053
Einkommen	−0,269***	−0,223***	**−0,292 bis −0,153**	**−0,203**
Ländliche Wohnregion (Ref. nichtländliche Wohnregion)	−0,008	−0,047	−0,139–0,043	−0,026
Onliner (Ref. Offliner)	−0,420***	−0,032	−0,314–0,234	−0,009
*Technikeinstellung*				
A: wahrgenommener adaptiver Nutzen	−0,191***	−0,103***	**−0,163 bis −0,036**	**−0,125**
B: technologiebezogenes Zielengagement	−0,127***	−0,042	−0,093–0,009	−0,053
C: wahrgenommene Sicherheit der Technologie	−0,146***	−0,061	−0,127–0,002	−0,066
*F*	17,945 (*p*: < 0,001)			
*Korrigiertes R*^*2*^	0,103			
*N (gültige)*	1335			

Abhängige Variable: subjektives digitales Exklusionsgefühl (SDE; Tab. 1) für Skalen der unabhängigen Variablen. Lineare Regression (Methode: Einschluss). *b* Regressionskoeffizient, *β* tandardisierter Regressionskoeffizient

Signifikanzniveaus: ***p < 0,001, **p < 0,01, *p < 0,05

### Gefühl des sozialen Drucks

Neben dem SDE konnte eine zusätzliche Aussage zum sozialen Druck, die neuesten technischen Errungenschaften nutzen zu müssen, in der Befragung bewertet werden. Die Aussage lautete: „Ich empfinde einen gewissen Druck, digitale Dienstleistungen nutzen zu müssen.“ Diese konnte anhand einer Skala von 1 „stimme gar nicht zu“ bis 5 „stimme voll und ganz zu“ bewertet werden. Über alle Altersgruppen ergibt sich ein Mittelwert von 3,00 (SD ± 1,29) oder anders ausgedrückt: Es stimmen 40,6 % der befragten Personen der Aussage eher oder voll und ganz zu. Der Altersgruppenvergleich zeigt ein ähnliches Muster wie bei dem Exklusionsempfinden. Personen ab 65 Jahren geben im Schnitt höhere Werte bei der Aussage an (Mittelwert: 3,24) als Personen unter 65 Jahren (Mittelwert: 2,91), was bedeutet, dass gerade ältere Personen in dieser Hinsicht sozialen Druck wahrnehmen.

## Diskussion

### Interpretation der Ergebnisse

Die Untersuchung des SDE zeigt auf, dass in der Schweizer Gesamtbevölkerung ab 18 Jahren eher zurückhaltende Exklusionsgefühle geäußert werden, wobei ein Teil (4,1 %) der Gesamtbevölkerung diese Exklusionsgefühle deutlich äußert. Unter den 65 Jahre alten und älteren Personen sind es 5,5 %, die hohe Werte aufweisen. Insofern lässt sich ein Unterschied zwischen der jüngeren (18 bis 64 Jahre) und älteren Bevölkerung (65+ Jahre) feststellen; dieser macht deutlich, dass Personen im Pensionsalter (65+) sich eher aus dem digitalen Alltag ausgeschlossen fühlen. Gleichzeitig zeigen die weiteren multivariaten Analysen, dass dieser Altersgruppenunterschied an Bedeutung verliert, wenn weitere standarddemografische Variablen und die Technikeinstellungen mitberücksichtigt werden. Im Regressionsmodell sind nur noch Einkommen und der wahrgenommene Nutzen in der Technikanwendung bedeutsam, womit Personen mit einem höheren Einkommen und einer positiven Technikeinstellung (die Technik verspricht einen hohen Nutzen) sich auch weniger digital ausgeschlossen fühlen.

Der Aspekt, dass sich gerade ältere Menschen mit einer eher negativen Einstellung zu neueren Technologien ausgeschlossen fühlen, konnte bereits in einer vorhergehenden Studie [15], in der nur Personen ab 65 Jahren berücksichtigt wurden, aufgezeigt werden. Daraus kann geschlossen werden, dass das kalendarische Alter das Gefühl der Ausgrenzung aus der Gesellschaft in Bezug auf die Techniknutzung nicht monokausal beeinflusst, sondern dass dieses mindestens durch andere Prädiktoren mitbestimmt ist. Dabei soll nicht unerwähnt bleiben, dass deskriptiv Altersgruppenunterscheide sichtbar wurden, die deutlich machen, dass das Gefühl der digital begründeten Ausgrenzung für einen Teil der älteren Personen real und bedeutsam ist. Gerade jene Personen, die hohe SDE haben, befinden sich in der Situation, dass ihnen die technischen Entwicklungen und die zunehmende Dominanz von digitalen Angeboten und Informationen das Gefühl geben, nicht dazuzugehören. Dies heißt z. B., dass gerade jene Personen, die z. B. während der COVID-19-Pandemie nicht die Möglichkeiten hatten, die Kontakte zu Freunden und Verwandten mithilfe digitaler Lösungen aufrechtzuerhalten, sich exkludiert fühlen könnten [14].

Auch wenn der digitale Graben zwischen Jung und Alt zunehmend geringer wird, zeigt die Studie, dass sich bereits heute ein Teil der älteren Bevölkerung ausgegrenzt fühlt und teilweise unter dem sozialen Druck steht, den Umgang mit den neusten Technologien erlernen zu müssen, um mitzuhalten. Das bedeutet potenziellen Stress hinsichtlich der Erfüllung (inter)subjektiver Kriterien, ein aktives Mitglied in der Gesellschaft zu sein [10, 12, 18]. Insbesondere diejenigen, die wenig bis gar nicht in der heutigen digitalen Welt vertreten sind, könnten sich zunehmend sozial exkludiert fühlen, wenn analoge Dienstleistungen und Kommunikationsmöglichkeiten wie ein bedienter Postschalter aufgrund von z. B. Sparmaßnahmen wegfallen, oder wenn etwa pandemiebedingte fehlende direkte Kontakte nicht durch digitale Lösungen kompensiert werden können [14]. Es besteht die Gefahr, dass sich diese älteren Menschen einem „Diktat des Digitalen“ [11] gegenübergestellt sehen, wenn eine Teilhabe in der heutigen Gesellschaft bedeutet, bestimmte Technologien nutzen zu müssen. Die „Ausgegrenzten“ könnten zu den Verlierern des technischen Fortschritts zählen und sich im Besonderen im Altersgruppenvergleich „abgehängt“ und „alt“ fühlen. Die aktuelle COVID-19-Pandemie hat sicherlich dieses Gefühl der digitalen Exklusion noch stärker sichtbar gemacht, jedoch kann mit den vorliegenden Daten kein Zeitvergleich geleistet werden; hier sind zukünftige Studien notwendig.

Daher sollte innerhalb der Gerontologie nicht mehr nur gefragt werden, ob die ältere Bevölkerungsgruppe „digital mit dabei ist oder nicht“, sondern wie sich eine Nichtteilnahme bzw. bedingte Teilnahme in einem SDE zeigt, und damit in der Frage, welche sozialen Folgen für das gesellschaftliche Teilhabegefühl bei älteren Menschen sich aus einer zunehmend technologisierten Alltagswelt ergeben können.

### Limitationen

Bei der durchgeführten Studie handelt es sich um eine Querschnittuntersuchung; Veränderungen innerhalb einer Person können daher nicht abgebildet werden. Für die weitere Forschung ist es wünschenswert, individuelle Daten im Längsschnitt, anhand derer die intraindividuellen Exklusionsgefühle beobachtet werden könnten, zu erheben. Die Studie wurde Ende 2019 durchgeführt, daher sind Vergleiche mit der COVID-19-Pandemie mit den vorliegenden Daten derzeit nicht möglich; neuere Daten werden sicherlich den Vergleich erlauben. Die Studie konnte Altersgruppenunterschiede aufzeigen. Jedoch sollten zukünftige Studien auch Kohorteneffekte und biografische Kontexte der befragten Personen berücksichtigen, damit neben dem kalendarischen Alter der biografische Hintergrund (z. B. berufsbedingte Vorerfahrungen mit der Techniknutzung) bei der Frage der Techniknutzung bzw. Nichtnutzung einbezogen wird. Die vorgelegten Daten beziehen sich nur auf die Schweiz. Daher sind direkte Übertragungen der Ergebnisse auf andere Länder nur für vergleichende Zwecke heranzuziehen; allfällige kulturelle Unterschiede sollten beachtet werden. Auch wurden keine persönlichen Kontextinformationen wie z. B. der Gesundheitszustand erhoben. Zudem wurden in die Befragung nur Personen in Privathaushalten inkludiert; es fehlt an Studien zu dieser Fragestellung in stationären Einrichtungen der Alterspflege [4, 5]. Auch konnten – trotz der Verwendung der drei Schweizer Landessprachen im Fragebogen – nicht Personen mit fehlenden Sprachkenntnissen erreicht werden.

## Fazit für die Praxis

- Obwohl ältere Personen immer mehr moderne Technologien nutzen, zeigt die Studie, dass ein Teil dieser Personen bereits heute befürchtet, den Anschluss an die Gesellschaft verloren zu haben, da sie die neuesten Technologien nicht ausreichend beherrschen oder zu beherrschen glauben.
- Neben älteren Personen fühlen sich auch junge Menschen teilweise aus der Gesellschaft ausgeschlossen, da sie technisch nicht mehr Schritt halten können. Unter ihnen sind es vorwiegend jene mit einer eher skeptischen Einstellung gegenüber der Technik.
- Die Zivilgesellschaft sollte für Schwierigkeiten älterer Menschen im Umgang mit Technik sensibilisiert sein, damit „Technikferne“ sich nicht aus dem Alltag ausgeschlossen fühlen.
